# Trends in malaria indicators after scale-up of community-based malaria management in Afghanistan

**DOI:** 10.1186/s12936-022-04174-x

**Published:** 2022-06-03

**Authors:** Sayed Daoud Mahmoodi, Abdul Alim Atarud, Ahmad Walid Sediqi, Sarah Gallalee, Willi McFarland, Temesgen Birara Aynie, Mohmmad Sami Nahzat, Hamida Hamid, Ghulam Qader Qader, Mohammad Shoaib Tamim, Ali Mirzazadeh

**Affiliations:** 1 United Nations Development Programme, Kabul, Afghanistan; 2 Institute for Global Health Sciences, University of California San Francisco, San Francisco, CA USA; 3 Department of Epidemiology and Biostatistics, University of California San Francisco, San Francisco, CA USA; 4 National Malaria Leishmania Control Programme, Ministry of Public Health, Kabul, Afghanistan; 5 Sustaining Technical and Analytical Resources (STAR) project, Public Health Institute (PHI), Kabul, Afghanistan

**Keywords:** Malaria, Community-based, Afghanistan, Diagnostic tests

## Abstract

**Background:**

The Community-Based Malaria Management (CBMM) strategy, introduced in 2013 and expanded to all health facilities and health posts in Afghanistan by 2016, aimed to deliver rapid diagnostic testing and more timely treatment to all communities nationwide. In this study, trends for several malaria outcome indicators were compared before and after the expansion of the CBMM strategy, using cross-sectional analysis of surveillance data.

**Methods:**

Generalized estimating equation (GEE) models with a Poisson distribution were used to assess trends of three key outcomes before (2012–2015) and after (2016–2019) CBMM expansion. These outcomes were annual malaria incidence rate (both all and confirmed malaria incidence), malaria death rate, and malaria test positivity rate. Additional variables assessed included annual blood examination rates (ABER) and malaria confirmation rate.

**Results:**

Average malaria incidence rates decreased from 13.1 before CBMM expansion to 10.0 per 1000 persons per year after CBMM expansion (P < 0.001). The time period after CBMM was expanded witnessed a 339% increase in confirmed malaria incidence as compared to the period before (IRR 3.39, 95% CI 2.18, 5.27; P < 0.001). In the period since the expansion of CBMM (2016–2019), overall malaria incidence rate declined by 19% each year (IRR 0.81, 95% CI 0.71,0.92; P = 0.001) and the malaria death rate declined by 85% each year (IRR 0.15, 95% CI 0.12, 0.20; P < 0.001). In comparing the before period to the after period, the ABER increased from 2.3 to 3.5 per 100 person/year, the malaria test positivity rate increased from 12.2 to 20.5%, and the confirmation rate increased from 21% before to 71% after CBMM.

**Conclusions:**

Afghanistan’s CBMM expansion to introduce rapid diagnostic tests and provide more timely treatment for malaria through all levels of care temporally correlates with significant improvement in multiple indicators of malaria control.

## Background

Worldwide, malaria is a major public health problem with 241 million new infections and 627,000 deaths annually [1]. Afghanistan, a country in the World Health Organization (WHO) Eastern Mediterranean Region, has relatively low transmission of malaria [2]. The Afghanistan National Malaria and Leishmania Control Programme reported 174,893 malaria cases and zero deaths in 2019, the lowest number that has ever been reported for the country. The two main species of malaria parasites in Afghanistan are *Plasmodium vivax* (98% of all cases) and *Plasmodium falciparum* (2%) [2].

In Afghanistan, malaria incidence rates vary by location. The variation results from differences in parasites, vectors, human population density, behaviours, ecological, high temperature, humidity and agriculture (rice cultivation), socio-economic conditions, and access to health services for detection and treatment of malaria. Nationally, 27% of the Afghan population lives in areas at high risk for malaria. Areas at high risk are defined as provinces and districts with annual parasite incidence (API) rate per 1000 persons at risk of 1 or above and test positivity rate (TPR) at 9% and above. Half (50%) of the population lives in areas at medium risk (API < 1, TPR < 9%), and the remaining 23% live in areas with low and very low risk of malaria transmission or its absence in malaria free areas [3]. In 2019, more than 93% of total malaria cases were reported from six provinces that border with Pakistan (Nangarhar, Laghman, Kunar, Nooristan, Khost, and Paktika) and one district of Kabul. Nangarhar is one highest endemic province in the country and accounted for more than 45% of total malaria cases and 35% of total *P. falciparum* cases [2].

Malaria diagnosis either by microscopy or rapid diagnostic tests is recommended by the WHO for all suspected malaria cases before starting the treatment. Early and accurate diagnosis is essential both for effective management of the disease, and for malaria surveillance and elimination strategies. In Afghanistan, the Community-Based Management of Malaria (CBMM) strategy was designed to progressively expand access to malaria diagnosis and effective anti-malarial treatment at non-diagnostic health facilities and community including health posts [4]. Malaria diagnosis using microscopy has been available in all hospitals and Comprehensive Health Centres (CHCs) of Afghanistan. Since 2013, the focus of the CBMM in Afghanistan has changed to specifically increase access to rapid diagnostic testing (RDT) and timely treatment at the community level in all malaria endemic and non-endemic areas of Afghanistan. The programme consists of two key modules; case management, vector control; CBMM was scaled up nationwide in 2016 with the support of the Global Fund. A main pillar of this revised strategy is introducing RDT in all health facilities, not only those providing diagnosis and treatment for malaria, and expanding screening of malaria to health posts to run community-based screening programs. In addition, the CBMM expanded the community-based malaria case management program using networks of community health workers (CHW) to reach all patients with suspected malaria at a level closer to the home. Since 2016, more than 30,000 CHWs were trained on malaria case management, RDT use and distribution of long-lasting insecticidal net (LLIN) to community through mass campaign. Other malaria commodities, including medicines, were supplied to health posts and health facilities without laboratory services. As a result, in 2017 more than 90% of CHW reported screening and referral of newly identified cases of malaria, and more than 50% reported providing counselling, chloroquine treatment for vivax malaria, and artemisinin-based combination therapy for suspected and confirmed falciparum malaria cases [5].

While the magnitude of the scale-up and shift in focus of the CBMM are encouraging, the effectiveness of the programme in Afghanistan has not yet been evaluated. In this study, trends in annual malaria incidence and death rates were assessed during two time periods, 4 years before the expansion of CBMM (2012–2015), and 4 years after expansion the CBMM program (2016–2019). Additional indicators of programme impact were also tracked. The scope of analysis included both national and subnational trends in Afghanistan.

## Methods

Data were extracted from the Malaria Leishmania Information System (MLIS) of the National Malaria Control Programme (NMCP) and Health Management Information System (HMIS). Data included clinical (diagnosed without a diagnostic test) and confirmed (diagnosed with a diagnostic test) malaria cases reported by approximately 2800 health facilities on a monthly basis. Patients were those with symptoms or diagnosis of malaria who visited health facilities, health posts and community member reached through outreach or mobile services. Data were initially collected on paper forms. The HMIS officers of non-governmental organizations (NGOs) and provincial malaria case managers checked the quality and completeness of the forms and entered them into the HMIS database. Hard and soft copies of collected data were shared with the provincial health directorate HMIS team on a monthly basis. The provincial HMIS and malaria officers reviewed and compiled the data and reported to the NMCP on a quarterly basis. Data were analysed and feedback provided to implementers on a quarterly basis. For this analysis, all data reported from 2012 to 2019 were used.

## Analysis

To assess trends in malaria before and after the expansion of the CBMM programme in Afghanistan, seven indicators were measured (Table 1). The descriptive analysis included the following indicators: the malaria incidence rate (both all and confirmed malaria) per 1000 persons per year, malaria death rates per 100,000 persons per year, malaria test positivity rate, annual blood examination rate per 100 per year (ABER) and the malaria confirmation rate. Reporting completeness during this time period was assessed to understand the reliability of the data.

**Table 1:** Indicators of malaria, Afghanistan, 2012–2019

Indicator	Numerator	Denominator
Malaria incidence rate (per 1,000 persons per year)	Number of reported (clinical and confirmed) malaria cases during the reporting year × 1000	Mid-year number of people at risk for malaria infection during the reporting year
Confirmed malaria incidence rate (per 1000 persons per year)	Number of confirmed malaria cases by microscopy or RDT during the reporting year × 1000	Mid-year number of people at risk for malaria infection during the reporting year
Malaria death rate (per 100,000 persons per year)	Number of in-patient malaria deaths during the reporting year × 100,000	Mid-year number of people at risk for malaria infection during the reporting year
Malaria test positivity rate (per 100 malaria tests per year)	Number of confirmed malaria cases by microscopy or RDT during the reporting year × 100	Total number of tests for malaria (RDT and microscopy) during the reporting year
Malaria confirmation rate (per 100 reported cases per year)	Number of malaria cases confirmed by microscopy or RDT during the reporting year × 100	Total reported malaria cases (clinical and confirmed)
Annual blood examination rate (per 100 population per year)	Number of persons receiving a parasitological test for malaria (microscopy or RDT) × 100	Population at risk (number of people living in areas where malaria transmission occurs)
Malaria reporting completeness (%)	Number of monthly malaria reports that were received from health facilities for the reporting year × 100	Number of all monthly malaria reports expected from health facilities for the reporting year

Data source for all indicators: Afghanistan Health Management Information System (HMIS), Malaria and Leishmaniasis Information System (MLIS); Years when data were available for all indicators: 2012–2019

Generalized estimating equation (GEE) models with a Poisson distribution were used to assess the differences in these indicator rates before (2012–2015) and after (2016–2019) CBMM was expanded (a binary predictor variable of before and after was used). Temporal trends during the before and after years were conducted by using GEE models by stratifying on time period and using year as a predictor variable. Analyses were conducted at the provincial level with all the provinces of Afghanistan included. Stata v.15. was used for statistical analysis and ArcGIS v.10.3.1 was used to create maps of average annual malaria incidence and average annual incidence of death due to malaria during the before and after periods.

## Results

**Fig. 1:**
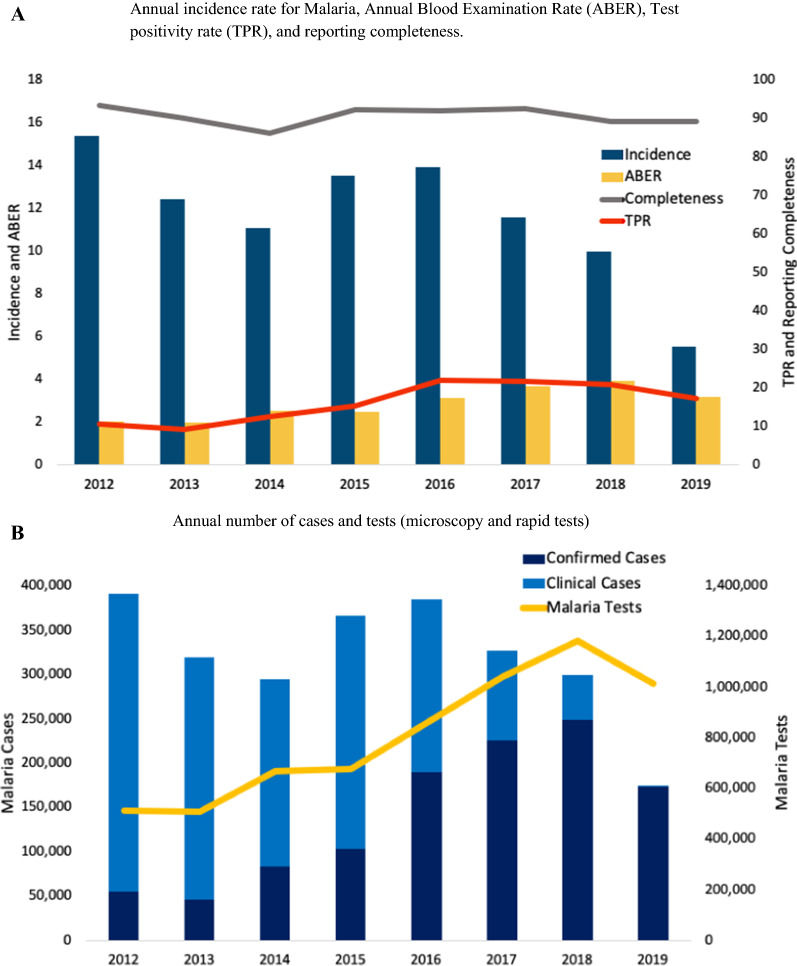
Several Malaria indicators in Afghanistan before (2012–15) and after (2016–2019) expansion the Rapid Testing for Malaria

Between 2012 and 2019, the total number of malaria cases (including clinical and confirmed) fell from 391,365 to 174,893. The overall malaria incidence rate declined from 15.4 to 5.5 per 1,000 per year and the malaria confirmation rate increased from 14 to 99% (Fig. 1A). The number of malaria cases that were confirmed by testing rose from 54,840 to 173,859; clinical cases declined 336,525 to 1034 (Fig. 1B). The malaria death rate fell from 0.1416 to 0 per 100,000 per year.

**Fig. 2:**
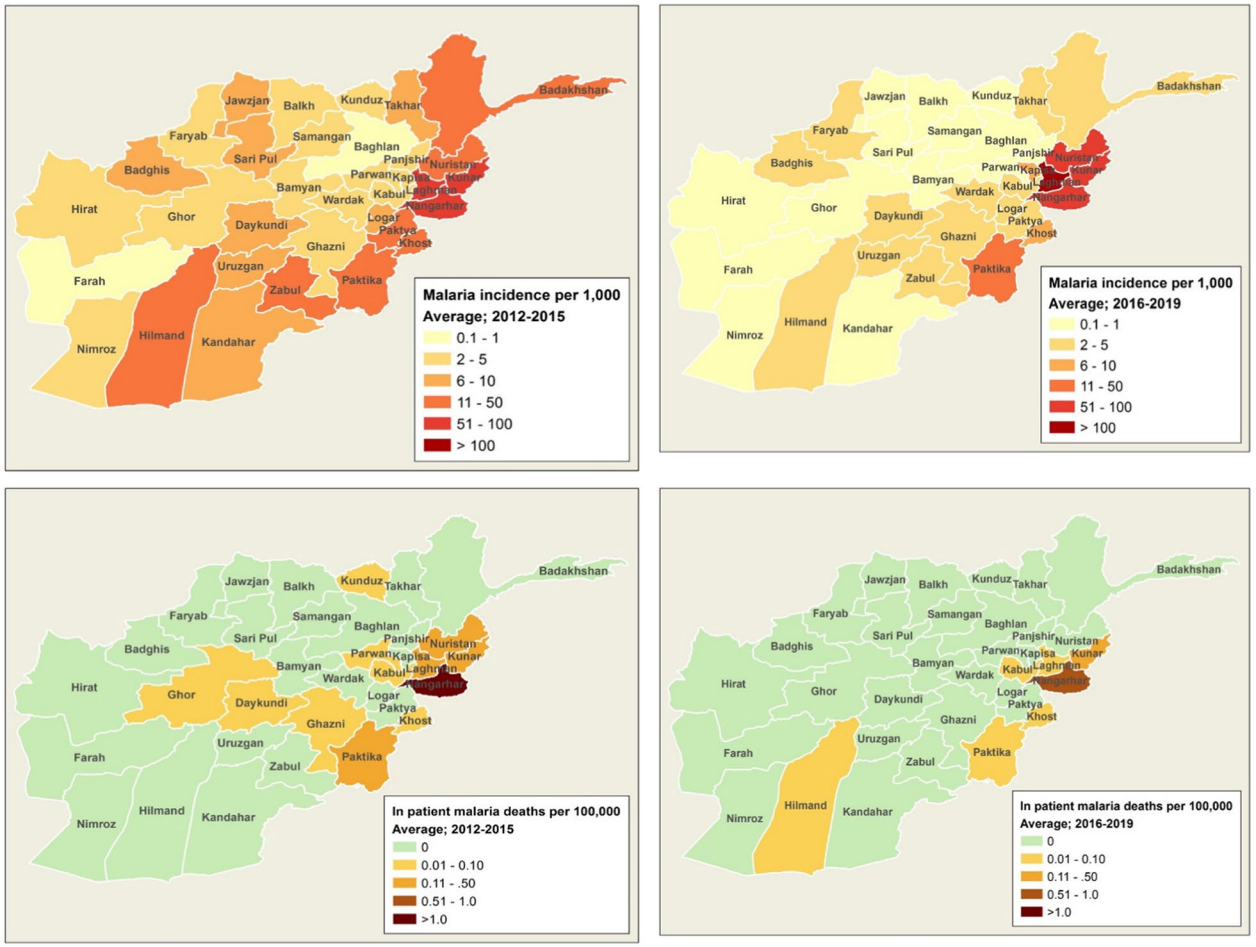
Malaria incidence and death rates due to malaria in Afghanistan before (2012–15; average of annual incidence) and after (2016–2019; average of annual incidence) expansion of CBMM

Table 2 presents annual malaria data and combined data for the two time periods based on the start of CBMM expansion (2012–15 vs. 2016–19). Between 2012 and 2015, the total number of tests conducted was 2,365,753. After the expansion of CBMM (2016–2019), the total number of tests conducted was 4,097,900 (Table 2; Fig. 1B). Meanwhile, average malaria incidence rates decreased from 13.1 before CBMM expansion to 10.1 per 1000 persons per year after CBMM expansion. The malaria death rates per 100,000 decreased from 0.1345 to 0.0493 for the years after CBMM expansion. The malaria test positivity rate increased 12.2–20.5%. The ABER increased from 2.3 to 3.5 per 100 per year. The malaria confirmation rate increased from 14% to 2012 to 99% in 2019. Annual malaria testing, incidence, and deaths are presented in Appendix 1 by province from 2012 to 2019 (Fig. 2). The average annual Malaria incidence and death rates in Afghanistan before (2012–15) and after (2016–2019) the expansion of CBMM are presented in Fig. 2. 

**Table 2:** Annual malaria data and indicators in Afghanistan from 2012 to 2019

Year	2012	2013	2014	2015	2016	2017	2018	2019	2012–15	2016–19
Population	25,427,322	25,740,700	26,588,632	27,101,365	27,657,145	28,227,323	30,075,018	31,575,018	26,214,505	29,383,626
Microscopy and rapid tests done for Malaria diagnosis	511,408	507,145	670,385	676,815	860,575	1,040,539	1,184,227	1,012,559	2,365,753	4,097,900
Rapid diagnostic tests done for Malaria	NA	NA	NA	NA	262,019	431,157	519,360	451,505	NA	1,664,041
*Plasmodium vivax* (PV) Malaria cases	53,609	43,842	77,937	98,357	180,729	216,064	239,762	170,746	273,745	807,301
*Plasmodium falciparum* (PF) Malaria cases	1231	2272	5983	5020	9430	10,111	8927	3113	14,506	31,581
Confirmed Malaria cases	54,840	46,114	83,920	103,377	190,159	226,175	248,689	173,859	288,251	838,882
Clinical Malaria cases	336,525	273,628	211,130	263,149	194,784	100,450	51,174	1,034	1,084,432	347,442
Reported malaria cases (clinical and confirmed)	391,365	319,742	295,050	366,526	384,943	326,625	299,863	174,893	1,372,683	1,186,324
Malaria Deaths	36	24	32	49	47	10	1	0	141	58
*Indicators of malaria*										
PV incidence rate per 1000 persons per year	2.11	1.7	2.93	3.63	6.53	7.65	7.97	5.41	2.61	6.87
PF incidence rate per 1000 persons per year	0.05	0.09	0.23	0.19	0.34	0.36	0.3	0.1	0.14	0.27
Malaria incidence rate (per 1000 persons per year)	15.4	12.4	11.1	13.5	13.9	11.6	10.0	5.5	13.1	10.1
Confirmed malaria incidence rates (per 1000 persons per year)	2.2	1.8	3.2	3.8	6.9	8.0	8.3	5.5	2.7	7.1
Malaria death rate (per 100,000 persons per year)	0.1416	0.0932	0.1204	0.1808	0.1699	0.0354	0.0033	0	0.1345	0.0493
Malaria test positivity rate (per 100 malaria tests per year)	10.7	9.1	12.5	15.3	22.1	21.7	21	17.2	12.2	20.5
Malaria confirmation rate (per 100 reported cases per year)	14	14	28	28	49	69	83	99	0.21	0.71
Annual blood examination rate (per 100 population per year)	2	2	2.5	2.5	3.1	3.7	3.9	3.2	2.3	3.5
Malaria reporting completeness (%)	93.4	90.1	86.0	92.3	92.0	92.6	89.1	89.3	90.4	90.8

Gy lines: Years of expansion of rapid diagnostic test for malaria

In the time period after CBMM expansion there was an 8% decrease in the malaria incidence rate as compared to the period before CBMM expansion (IRR 0.92, P = 0.692) (Table 3). For the time period after CBMM expansion, the confirmed malaria incidence rate increased 339% as compared to the period before CBMM was expanded (IRR 3.39, P < 0.001). There was a 65% decrease in the malaria death rate in the period after the expansion of CBMM compared to the period before (IRR 0.35, P < 0.001).

**Table 3:** Comparison of the period before CBMM and the period after the expansion of CBMM for malaria in Afghanistan

Predictor Variable: Binary before and after CBMM	Unadjusted univariable model			
	IRR	95% CI		P-value
Outcome: malaria incidence	0.92	0.62	1.38	0.692
Outcome: confirmed malaria incidence	3.39	2.18	5.27	**< 0.001**
Outcome: malaria death incidence	0.35	0.28	0.44	**< 0.001**

IRR: Incidence Rate Ratio; CI: Confidential Interval

In examining only, the period since the expansion of CBMM (2016–2019), the overall malaria incidence rate declined by 19% each year (IRR 0.81, P = 0.001). The confirmed malaria incidence rate declined by 2% each year (IRR:0.98, P = 0.840). Malaria death incidence declined by 85% each year (IRR 0.15, P < 0.001) (Table 4).

**Table 4:** Average annual change in malaria outcomes before (2012-15) and after (2016–2019) expansion of CBMM for Malaria in Afghanistan

Predictor Variable: Year	Before CBMM expansion (2012–2015)				After CBMM expansion (2016–2019)			
	IRR	95% CI		P-value	IRR	95% CI		P-value
Outcome: malaria incidence	0.97	0.89	1.05	0.431	0.81	0.71	0.92	**0.001**
Outcome: confirmed malaria incidence	1.29	1.09	1.53	**0.003**	0.98	0.85	1.14	0.835
Outcome: malaria death incidence	1.13	0.88	1.44	0.344	0.15	0.12	0.20	**< 0.001**

IRR: Incidence Rate Ratio; CI: Confidential Interval

## Discussion

The malaria trend analysis revealed several encouraging outcomes for malaria control in Afghanistan following the scale-up of the CBMM strategy. In line with the expansion of RDT, there was an increase in the number of suspected cases that received parasitological testing in a health facility and at community levels. During the period since this expansion, the malaria incidence rate and malaria death rate declined. The magnitude of the decline in incidence is remarkable - from 15.5 to 5.5 per 1000 persons/year between 2012 and 2019. The malaria deaths rate declined from 0.1416 to 0 per 100,000 persons per year for the same periods. Additionally, number of confirmed malaria cases increased following the expansion of RDT and the number of clinical cases decreased during the period. The ABER have increased, leading to a confirmation rate of nearly 100%.

The study results are similar to positive outcomes of other community-based malaria control models. A systematic review conducted in 2019 investigated the impact of community-delivered models (namely, Integrated Community Case Management and Home Management of Malaria) on coverage and malaria outcomes compared to non-community-delivered models [6]. The result of meta-analysis indicated that the implementation of community-delivered models improved malaria-attributed mortality. Community-delivered models also reduced the risk of parasitaemia from 25 to 70% compared to non-community-delivered models [6].

There were four limitations in the study and analysis. First, surveillance and health system data were used which meant authors were not fully able to assess quality (however, there was very high reporting completeness throughout the study period). Second, data were reported as aggregated and individual characteristics such as gender, age, and other personal and behaviour data were not available. Studying the potential associations between malaria and these characteristics will help target future interventions towards malaria elimination. Third, the surveillance data did not include most of the cases, which were diagnosed or received treatment in private health sectors. It is also unclear how use of the private health sector changed over time. Lastly, treatment data were not reported to the surveillance system and, therefore, it was not possible to assess trends in this important indicator.

There are also potential confounders that may explain or partially explain the differences witnessed in malaria indicators during the before versus after CBMM scale-up. These include vector control measures, the Basic Package of Health Services (BPHS), the Essential Package of Health Services (EPHS) and strengthening of malaria surveillance, Malaria Leishmania Information System (MLIS). The diagnosis and treatment of malaria has been integrated into BPHS and EPHS services, with malaria diagnosis and treatment (including microscopy and anti-malarial therapy) provided from health post level up to regional hospitals and provided malaria reports on monthly basis. Additionally, since expansion of CBMM after 2016, approximately 6,015,826 long-lasting insecticide nets (LLIN) have been distributed to targeted provinces. The LLIN distribution programme ensured 100% operational coverage (i.e., all target provinces and districts were covered through mass distribution campaigns and through continuous distribution at antenatal clinics). The programme sought to improve coverage and accessibility for at-risk populations, including pregnant women and children. Ecological factors such as changes in temperature or rainfall, variables that could influence malaria transmission in Afghanistan were not assessed.

The trend analysis for the period after CBMM expansion shows that most of the targets of Afghanistan’s National Strategic Plan for Malaria 2018–22 are on track to being met. The plan aims to reduce malaria incidence by 73% at the national level compared with 2016. Between 2016 and 2019, the number of reported malaria cases were reduced from 385,015 to 174,893 (55%). The proportion of confirmed malaria cases increased to 99% in 2019 compared to the baseline 49% in 2016. Nonetheless, 12 provinces remain at high risk for malaria with reported annual parasite incidence rates per 1000 persons at 1 and above and test positivity rate at 9% and above.

## Conclusions

In summary, the CBMM expansion which introduced rapid diagnostic tests for malaria to many primary care settings correlated with significant increase in the number of confirmed cases, while also being correlated with significant reduction in annual malaria incidence and death rates. Use of RDTs for the diagnosis of malaria could be best applied as a tool at the community level to facilitate the early treatment of malaria in settings where microscopy services are not available. The data and the study results corroborate similar studies that recommend community-based interventions as best practices for malaria control, especially in resource-limited settings.

## Data Availability

All details of data (case numbers) that we used for our analysis are presented in Table 2 and Appendix 1.

## Appendix 1

Province wise annual malaria testing, incidence and deaths in Afghanistan from 2012 to 2019.

**Table:**

Province	Population	Total microscopy and rapid tests for Malaria diagnosis	*Plasmodium vivax* (PV) Malaria cases	PV incidence rate per 1000 persons per year	*Plasmodium falciparum* (PF) Malaria cases	PF incidence rate per 1000 persons per year	Total Confirmed Malaria cases	Total clinical malaria cases	Reported malaria cases (clinical and confirmed)	Malaria test positivity rate (per 100 malaria tests per year)	Malaria confirmation rate (per 100 reported cases per year)	Malaria incidence rate (per 1000 persons per year)	Annual blood examination rate (per 100 population per year)	Total malaria deaths
*Badakhshan*														
2012	889,700	17,646	1154	1.30	4	0.004	1158	13,573	14,731	6.6%	7.9%	16.56	1.98%	0
2013	919,900	14,408	1140	1.24	4	0.004	1144	10,592	11,736	7.9%	9.7%	12.76	1.57%	0
2014	935,327	23,665	2261	2.42	239	0.256	2500	5769	8269	10.6%	30.2%	8.84	2.53%	0
2015	950,953	20,646	1810	1.90	77	0.081	1887	5636	7523	9.1%	25.1%	7.91	2.17%	0
2016	966,789	12,104	1201	1.24	22	0.023	1223	4733	5956	10.1%	20.5%	6.16	1.25%	0
2017	982,835	17,078	1110	1.13	10	0.010	1120	526	1646	6.6%	68.0%	1.67	1.74%	0
2018	1,017,499	15,114	731	0.72	6	0.006	737	95	832	4.9%	88.6%	0.82	1.49%	0
2019	1,035,658	13,435	407	0.39	23	0.022	430	0	430	3.2%	100.0%	0.42	1.30%	0
*Badghis*														
2012	464,100	1682	27	0.06	0	0.000	27	6666	6693	1.6%	0.4%	14.42	0.36%	0
2013	479,800	1997	28	0.06	4	0.008	32	3095	3127	1.6%	1.0%	6.52	0.42%	0
2014	487,838	955	13	0.03	2	0.004	15	3150	3165	1.6%	0.5%	6.49	0.20%	0
2015	495,958	1042	18	0.04	2	0.004	20	3739	3759	1.9%	0.5%	7.58	0.21%	0
2016	504,185	10,356	3228	6.40	197	0.391	3425	2225	5650	33.1%	60.6%	11.21	2.05%	0
2017	512,518	5476	418	0.82	12	0.023	430	1590	2020	7.9%	21.3%	3.94	1.07%	0
2018	530,574	6545	23	0.04	2	0.004	25	195	220	0.4%	11.4%	0.41	1.23%	0
2019	540,009	4518	12	0.02	1	0.002	13	0	13	0.3%	100.0%	0.02	0.84%	0
*Baghlan*														
2012	848,500	5567	18	0.02	0	0.000	18	646	664	0.3%	2.7%	0.78	0.66%	0
2013	855,400	3246	21	0.02	3	0.004	24	347	371	0.7%	6.5%	0.43	0.38%	0
2014	894,838	7906	8	0.01	1	0.001	9	543	552	0.1%	1.6%	0.62	0.88%	0
2015	910,784	5451	31	0.03	5	0.005	36	95	131	0.7%	27.5%	0.14	0.60%	0
2016	926,969	5297	69	0.07	10	0.011	79	25	104	1.5%	76.0%	0.11	0.57%	0
2017	946,394	10,799	122	0.13	8	0.008	130	19	149	1.2%	87.2%	0.16	1.14%	0
2018	977,297	10,147	70	0.07	1	0.001	71	17	88	0.7%	80.7%	0.09	1.04%	0
2019	995,814	9218	52	0.05	3	0.003	55	0	55	0.6%	100.0%	0.06	0.93%	0
*Balkh*														
2012	1,219,200	3431	154	0.13	0	0.000	154	3583	3737	4.5%	4.1%	3.07	0.28%	0
2013	1,318,000	3680	138	0.10	4	0.003	142	3734	3876	3.9%	3.7%	2.94	0.28%	0
2014	1,298,247	11,520	153	0.12	65	0.050	218	1187	1405	1.9%	15.5%	1.08	0.89%	0
2015	1,325,659	11,261	155	0.12	25	0.019	180	1457	1637	1.6%	11.0%	1.23	0.85%	0
2016	1,353,626	3867	12	0.01	82	0.061	94	1137	1231	2.4%	7.6%	0.91	0.29%	0
2017	1,382,155	5942	67	0.05	5	0.004	72	559	631	1.2%	11.4%	0.46	0.43%	0
2018	1,442,847	6412	56	0.04	10	0.007	66	843	909	1.0%	7.3%	0.63	0.44%	0
2019	1,475,649	8627	59	0.04	1	0.001	60	0	60	0.7%	100.0%	0.04	0.58%	0
*Bamyan*														
2012	418,500	680	27	0.06	0	0.000	27	833	860	4.0%	3.1%	2.05	0.16%	0
2013	432,700	804	34	0.08	7	0.016	41	691	732	5.1%	5.6%	1.69	0.19%	0
2014	439,899	952	58	0.13	9	0.020	67	578	645	7.0%	10.4%	1.47	0.22%	0
2015	447,218	2281	27	0.06	16	0.036	43	673	716	1.9%	6.0%	1.60	0.51%	0
2016	427,067	2299	167	0.39	357	0.836	524	312	836	22.8%	62.7%	1.96	0.54%	0
2017	462,144	1940	9	0.02	0	0.000	9	121	130	0.5%	6.9%	0.28	0.42%	0
2018	478,424	1660	49	0.10	4	0.008	53	5	58	3.2%	91.4%	0.12	0.35%	0
2019	486,928	707	19	0.04	1	0.002	20	0	20	2.8%	100.0%	0.04	0.15%	0
*Daykundi*														
2012	431,300	2097	82	0.19	14	0.032	96	2913	3009	4.6%	3.2%	6.98	0.49%	1
2013	378,900	2594	47	0.12	4	0.011	51	1956	2007	2.0%	2.5%	5.30	0.68%	0
2014	417,476	1798	40	0.10	10	0.024	50	2029	2079	2.8%	2.4%	4.98	0.43%	0
2015	424,339	1633	34	0.08	10	0.024	44	1756	1800	2.7%	2.4%	4.24	0.38%	0
2016	468,178	2818	132	0.28	66	0.141	198	2493	2691	7.0%	7.4%	5.75	0.60%	0
2017	493,634	4376	52	0.11	19	0.038	71	1374	1445	1.6%	4.9%	2.93	0.89%	0
2018	544,788	3434	124	0.23	5	0.009	129	853	982	3.8%	13.1%	1.80	0.63%	0
2019	507,610	4845	15	0.03	1	0.002	16	0	16	0.3%	100.0%	0.03	0.95%	0
*Farah*														
2012	480,500	1221	40	0.08	2	0.004	42	438	480	3.4%	8.8%	1.00	0.25%	0
2013	490,600	1186	15	0.03	5	0.010	20	337	357	1.7%	5.6%	0.73	0.24%	0
2014	498,951	658	17	0.03	1	0.002	18	310	328	2.7%	5.5%	0.66	0.13%	0
2015	507,405	815	13	0.03	7	0.014	20	381	401	2.5%	5.0%	0.79	0.16%	0
2016	515,973	3143	33	0.06	5	0.010	38	258	296	1.2%	12.8%	0.57	0.61%	0
2017	524,657	4942	110	0.21	2	0.004	112	311	423	2.3%	26.5%	0.81	0.94%	0
2018	543,237	5096	84	0.15	0	0.000	84	23	107	1.6%	78.5%	0.20	0.94%	0
2019	553,058	1899	25	0.05	1	0.002	26	0	26	1.4%	100.0%	0.05	0.34%	0
*Faryab*														
2012	931,800	2989	31	0.03	1	0.001	32	4471	4503	1.1%	0.7%	4.83	0.32%	0
2013	964,600	3527	26	0.03	3	0.003	29	3901	3930	0.8%	0.7%	4.07	0.37%	0
2014	981,197	5136	914	0.93	98	0.100	1012	2898	3910	19.7%	25.9%	3.98	0.52%	0
2015	998,147	3808	0	0.00	1	0.001	1	2461	2462	0.0%	0.0%	2.47	0.38%	0
2016	1,015,335	3966	14	0.01	0	0.000	14	2559	2573	0.4%	0.5%	2.53	0.39%	0
2017	1,032,765	7098	3	0.00	7	0.007	10	1099	1109	0.1%	0.9%	1.07	0.69%	0
2018	1,069,540	3747	30	0.03	0	0.000	30	472	502	0.8%	6.0%	0.47	0.35%	0
2019	1,089,228	3799	12	0.01	1	0.001	13	1	14	0.3%	92.9%	0.01	0.35%	0
*Ghazni*														
2012	1,149,400	10,086	1179	1.03	61	0.053	1240	4109	5349	12.3%	23.2%	4.65	0.88%	0
2013	1,188,600	14,258	1307	1.10	41	0.034	1348	4001	5349	9.5%	25.2%	4.50	1.20%	1
2014	1,240,437	16,606	1329	1.07	83	0.067	1412	3241	4653	8.5%	30.3%	3.75	1.34%	0
2015	1,228,831	17,392	832	0.68	80	0.065	912	2723	3635	5.2%	25.1%	2.96	1.42%	0
2016	1,249,376	22,488	841	0.67	67	0.054	908	2297	3205	4.0%	28.3%	2.57	1.80%	0
2017	1,270,192	17,949	959	0.76	85	0.067	1044	961	2005	5.8%	52.1%	1.58	1.41%	0
2018	1,315,041	8154	610	0.46	23	0.017	633	359	992	7.8%	63.8%	0.75	0.62%	0
2019	1,338,597	9905	750	0.56	129	0.096	879	1	880	8.9%	99.9%	0.66	0.74%	0
*Ghor*														
2012	646,300	361	19	0.03	34	0.053	53	1817	1870	14.7%	2.8%	2.89	0.06%	1
2013	668,000	585	15	0.02	29	0.043	44	1586	1630	7.5%	2.7%	2.44	0.09%	0
2014	679,085	415	5	0.01	22	0.032	27	1407	1434	6.5%	1.9%	2.11	0.06%	0
2015	690,296	339	7	0.01	29	0.042	36	1004	1040	10.6%	3.5%	1.51	0.05%	0
2016	701,653	1332	23	0.03	5	0.007	28	540	568	2.1%	4.9%	0.81	0.19%	0
2017	713,158	4755	39	0.05	11	0.015	50	485	535	1.1%	9.3%	0.75	0.67%	0
2018	738,224	3360	43	0.06	4	0.005	47	291	338	1.4%	13.9%	0.46	0.46%	0
2019	751,254	4637	13	0.02	0	0.000	13	0	13	0.3%	100.0%	0.02	0.62%	0
*Hilmand*														
2012	864,600	8643	314	0.36	32	0.037	346	11,296	11,642	4.0%	3.0%	13.47	1.00%	0
2013	867,600	10,776	375	0.43	63	0.073	438	12,939	13,377	4.1%	3.3%	15.42	1.24%	0
2014	909,395	14,840	204	0.22	104	0.114	308	12,845	13,153	2.1%	2.3%	14.46	1.63%	0
2015	924,711	13,146	106	0.11	11	0.012	117	12,631	12,748	0.9%	0.9%	13.79	1.42%	0
2016	894,805	11,746	106	0.12	13	0.015	119	7611	7730	1.0%	1.5%	8.64	1.31%	1
2017	938,184	21,474	192	0.20	32	0.034	224	8922	9146	1.0%	2.4%	9.75	2.29%	0
2018	1,395,514	24,637	101	0.07	10	0.007	111	978	1089	0.5%	10.2%	0.78	1.77%	0
2019	1,420,682	15,466	51	0.04	3	0.002	54	0	54	0.3%	100.0%	0.04	1.09%	0
*Hirat*														
2012	1,744,700	1927	9	0.01	0	0.000	9	5373	5382	0.5%	0.2%	3.08	0.11%	0
2013	1,816,100	2285	3	0.00	0	0.000	3	2791	2794	0.1%	0.1%	1.54	0.13%	0
2014	1,852,790	5567	24	0.01	1	0.001	25	1713	1738	0.4%	1.4%	0.94	0.30%	0
2015	1,890,202	6593	11	0.01	1	0.001	12	1325	1337	0.2%	0.9%	0.71	0.35%	0
2016	1,928,327	3295	12	0.01	3	0.002	15	947	962	0.5%	1.6%	0.50	0.17%	0
2017	1,967,180	9607	4	0.00	17	0.009	21	196	217	0.2%	9.7%	0.11	0.49%	0
2018	2,050,514	7736	12	0.01	0	0.000	12	117	129	0.2%	9.3%	0.06	0.38%	0
2019	2,095,117	4681	19	0.01	2	0.001	21	0	21	0.4%	100.0%	0.01	0.22%	0
*Jawzjan*														
2012	503,100	2263	20	0.04	0	0.000	20	3264	3284	0.9%	0.6%	6.53	0.45%	0
2013	521,400	2462	6	0.01	15	0.029	21	3967	3988	0.9%	0.5%	7.65	0.47%	0
2014	530,751	1957	11	0.02	4	0.008	15	1675	1690	0.8%	0.9%	3.18	0.37%	0
2015	540,255	2790	24	0.04	8	0.015	32	3001	3033	1.1%	1.1%	5.61	0.52%	0
2016	549,900	1738	3	0.01	5	0.009	8	1169	1177	0.5%	0.7%	2.14	0.32%	0
2017	559,691	2243	3	0.01	17	0.030	20	447	467	0.9%	4.3%	0.83	0.40%	0
2018	579,833	3196	17	0.03	0	0.000	17	21	38	0.5%	44.7%	0.07	0.55%	0
2019	590,866	6700	13	0.02	0	0.000	13	0	13	0.2%	100.0%	0.02	1.13%	0
* Kabul *														
2012	3,818,700	23,300	2013	0.53	20	0.005	2033	10,352	12,385	8.7%	16.4%	3.24	0.61%	1
2013	4,086,500	23,568	1133	0.28	74	0.018	1207	7441	8648	5.1%	14.0%	2.12	0.58%	1
2014	4,227,261	28,007	2013	0.48	37	0.009	2050	7971	10,021	7.3%	20.5%	2.37	0.66%	0
2015	4,372,977	30,343	3394	0.78	81	0.019	3475	9769	13,244	11.5%	26.2%	3.03	0.69%	1
2016	4,523,718	53,469	10,844	2.40	257	0.057	11,101	11,628	22,729	20.8%	48.8%	5.02	1.18%	5
2017	4,679,648	58,254	13,468	2.88	269	0.057	13,737	4678	18,415	23.6%	74.6%	3.94	1.24%	2
2018	4,860,880	63,631	11,208	2.31	180	0.037	11,388	3799	15,187	17.9%	75.0%	3.12	1.31%	1
2019	5,029,850	43,034	5906	1.17	126	0.025	6032	832	6864	14.0%	87.9%	1.36	0.86%	0
*Kandahar*														
2012	1,127,000	6510	134	0.12	10	0.009	144	12,154	12,298	2.2%	1.2%	10.91	0.58%	0
2013	1,119,000	6215	99	0.09	5	0.004	104	7935	8039	1.7%	1.3%	7.18	0.56%	0
2014	1,200,929	5613	70	0.06	1	0.001	71	6548	6619	1.3%	1.1%	5.51	0.47%	0
2015	1,226,593	7088	150	0.12	1	0.001	151	4859	5010	2.1%	3.0%	4.08	0.58%	0
2016	1,193,020	12,086	88	0.07	2	0.002	90	2908	2998	0.7%	3.0%	2.51	1.01%	0
2017	1,279,520	11,921	101	0.08	63	0.049	164	242	406	1.4%	40.4%	0.32	0.93%	0
2018	1,351,169	12,122	125	0.09	18	0.013	143	379	522	1.2%	27.4%	0.39	0.90%	0
2019	1,368,036	17,490	338	0.25	19	0.014	357	1	358	2.0%	99.7%	0.26	1.28%	0
*Kapisa*														
2012	413,000	5369	388	0.94	1	0.002	389	1594	1983	7.2%	19.6%	4.80	1.30%	0
2013	426,800	7527	275	0.64	1	0.002	276	1120	1396	3.7%	19.8%	3.27	1.76%	0
2014	433,867	5148	109	0.25	0	0.000	109	720	829	2.1%	13.1%	1.91	1.19%	0
2015	441,010	5855	140	0.32	0	0.000	140	653	793	2.4%	17.7%	1.80	1.33%	0
2016	448,245	8768	763	1.70	39	0.087	802	2403	3205	9.1%	25.0%	7.15	1.96%	0
2017	455,574	12,061	2091	4.59	49	0.108	2140	1152	3292	17.7%	65.0%	7.23	2.65%	0
2018	471,574	14,229	1608	3.41	12	0.025	1620	75	1695	11.4%	95.6%	3.59	3.02%	0
2019	479,875	11,687	1098	2.29	8	0.017	1106	0	1106	9.5%	100.0%	2.30	2.44%	0
*Khost*														
2012	537,800	10,861	1610	2.99	61	0.113	1671	10,461	12,132	15.4%	13.8%	22.56	2.02%	0
2013	556,000	14,161	1298	2.33	121	0.218	1419	9018	10,437	10.0%	13.6%	18.77	2.55%	1
2014	565,211	26,224	1560	2.76	286	0.506	1846	5207	7053	7.0%	26.2%	12.48	4.64%	1
2015	574,582	19,670	1435	2.50	492	0.856	1927	6445	8372	9.8%	23.0%	14.57	3.42%	0
2016	584,075	23,215	2473	4.23	349	0.598	2822	4292	7114	12.2%	39.7%	12.18	3.97%	1
2017	593,691	24,731	3600	6.06	407	0.686	4007	1675	5682	16.2%	70.5%	9.57	4.17%	0
2018	614,584	23,897	2864	4.66	106	0.172	2970	406	3376	12.4%	88.0%	5.49	3.89%	0
2019	625,473	20,294	1778	2.84	21	0.034	1799	1	1800	8.9%	99.9%	2.88	3.24%	0
*Kunar*														
2012	421,700	40,847	7464	17.70	200	0.474	7664	33,376	41,040	18.8%	18.7%	97.32	9.69%	0
2013	436,000	39,298	5354	12.28	173	0.397	5527	39,103	44,630	14.1%	12.4%	102.36	9.01%	0
2014	443,272	65,356	12,534	28.28	1277	2.881	13,811	23,198	37,009	21.1%	37.3%	83.49	14.74%	3
2015	450,652	55,648	12,150	26.96	308	0.683	12,458	32,670	45,128	22.4%	27.6%	100.14	12.35%	4
2016	440,231	67,782	19,235	43.69	914	2.076	20,149	28,522	48,671	29.7%	41.4%	110.56	15.40%	3
2017	465,706	115,289	37,373	80.25	1235	2.652	38,608	13,023	51,631	33.5%	74.8%	110.87	24.76%	1
2018	482,115	132,366	40,427	83.85	1119	2.321	41,546	6348	47,894	31.4%	86.7%	99.34	27.46%	0
2019	490,690	97,434	30,015	61.17	141	0.287	30,156	0	30,156	31.0%	100.0%	61.46	19.86%	0
*Kunduz*														
2012	935,600	11,155	123	0.13	0	0.000	123	5028	5151	1.1%	2.4%	5.51	1.19%	0
2013	972,200	9260	53	0.05	0	0.000	53	3480	3533	0.6%	1.5%	3.63	0.95%	0
2014	990,937	9437	46	0.05	6	0.006	52	1001	1053	0.6%	4.9%	1.06	0.95%	1
2015	1,010,037	6895	21	0.02	0	0.000	21	494	515	0.3%	4.1%	0.51	0.68%	0
2016	961,309	7123	54	0.06	4	0.004	58	344	402	0.8%	14.4%	0.42	0.74%	0
2017	1,049,249	8284	123	0.12	6	0.006	129	377	506	1.6%	25.5%	0.48	0.79%	0
2018	1,091,116	11,793	120	0.11	0	0.000	120	29	149	1.0%	80.5%	0.14	1.08%	0
2019	1,113,676	8881	93	0.08	0	0.000	93	0	93	1.0%	100.0%	0.08	0.80%	0
*Laghman*														
2012	417,200	37,619	4129	9.90	70	0.168	4199	30,394	34,593	11.2%	12.1%	82.92	9.02%	0
2013	431,200	37,810	2142	4.97	49	0.114	2191	24,334	26,525	5.8%	8.3%	61.51	8.77%	0
2014	438,346	50,427	8171	18.64	800	1.825	8971	23,292	32,263	17.8%	27.8%	73.60	11.50%	2
2015	445,588	78,359	17,784	39.91	902	2.024	18,686	37,723	56,409	23.8%	33.1%	126.59	17.59%	0
2016	445,238	169,476	64,194	144.18	3240	7.277	67,434	34,473	101,907	39.8%	66.2%	228.88	38.06%	1
2017	460,352	130,626	45,363	98.54	2789	6.058	48,152	21,184	69,336	36.9%	69.4%	150.62	28.38%	0
2018	476,537	167,947	53,724	112.74	3923	8.232	57,647	11,940	69,587	34.3%	82.8%	146.03	35.24%	0
2019	484,952	159,817	38,792	79.99	1173	2.419	39,965	177	40,142	25.0%	99.6%	82.78	32.96%	0
*Logar*														
2012	367,000	3008	285	0.78	6	0.016	291	1638	1929	9.7%	15.1%	5.26	0.82%	0
2013	379,400	2701	157	0.41	18	0.047	175	1213	1388	6.5%	12.6%	3.66	0.71%	0
2014	385,638	5128	851	2.21	27	0.070	878	1056	1934	17.1%	45.4%	5.02	1.33%	0
2015	392,045	4528	542	1.38	35	0.089	577	1896	2473	12.7%	23.3%	6.31	1.15%	0
2016	398,535	8510	1242	3.12	62	0.156	1304	1435	2739	15.3%	47.6%	6.87	2.14%	0
2017	405,109	11,846	1822	4.50	19	0.047	1841	580	2421	15.5%	76.0%	5.98	2.92%	0
2018	419,377	13,952	1627	3.88	26	0.062	1653	108	1761	11.8%	93.9%	4.20	3.33%	0
2019	426,821	12,110	694	1.63	6	0.014	700	0	700	5.8%	100.0%	1.64	2.84%	0
*Nangarhar*														
2012	1,409,600	244,604	29,108	20.65	517	0.367	29,625	116,035	145,660	12.1%	20.3%	103.33	17.35%	30
2013	1,462,600	236,080	25,217	17.24	1441	0.985	26,658	80,157	106,815	11.3%	25.0%	73.03	16.14%	18
2014	1,489,787	279,057	41,554	27.89	2542	1.706	44,096	63,032	107,128	15.8%	41.2%	71.91	18.73%	24
2015	1,517,388	274,610	53,087	34.99	2582	1.702	55,669	97,207	152,876	20.3%	36.4%	100.75	18.10%	45
2016	1,545,448	315,613	67,114	43.43	3187	2.062	70,301	64,644	134,945	22.3%	52.1%	87.32	20.42%	35
2017	1,573,973	416,333	92,948	59.05	3951	2.510	96,899	25,208	122,107	23.3%	79.4%	77.58	26.45%	7
2018	1,635,872	495,795	105,650	64.58	2405	1.470	108,055	12,731	120,786	21.8%	89.5%	73.84	30.31%	0
2019	1,668,481	423,073	74,825	44.85	1093	0.655	75,918	8	75,926	17.9%	100.0%	45.51	25.36%	0
*Nimroz*														
2012	147,700	83	1	0.01	0	0.000	1	382	383	1.2%	0.3%	2.59	0.06%	0
2013	152,800	82	2	0.01	0	0.000	2	307	309	2.4%	0.6%	2.02	0.05%	0
2014	162,135	81	1	0.01	0	0.000	1	241	242	1.2%	0.4%	1.49	0.05%	0
2015	164,978	197	1	0.01	5	0.030	6	246	252	3.0%	2.4%	1.53	0.12%	0
2016	161,033	770	5	0.03	8	0.050	13	105	118	1.7%	11.0%	0.73	0.48%	0
2017	170,790	1048	6	0.04	0	0.000	6	94	100	0.6%	6.0%	0.59	0.61%	0
2018	176,898	1054	4	0.02	0	0.000	4	68	72	0.4%	5.6%	0.41	0.60%	0
2019	180,200	1664	9	0.05	0	0.000	9	0	9	0.5%	100.0%	0.05	0.92%	0
*Nuristan*														
2012	138,600	5109	423	3.05	23	0.166	446	3220	3666	8.7%	12.2%	26.45	3.69%	0
2013	143,200	4797	355	2.48	26	0.182	381	3412	3793	7.9%	10.0%	26.49	3.35%	0
2014	145,574	6290	647	4.44	19	0.131	666	3040	3706	10.6%	18.0%	25.46	4.32%	1
2015	147,967	10,134	1725	11.66	43	0.291	1768	4332	6100	17.4%	29.0%	41.23	6.85%	0
2016	150,391	14,529	3732	24.82	134	0.891	3866	2714	6580	26.6%	58.8%	43.75	9.66%	0
2017	152,845	22,795	7033	46.01	247	1.616	7280	4891	12,171	31.9%	59.8%	79.63	14.91%	0
2018	158,211	31,952	11,687	73.87	432	2.731	12,119	5100	17,219	37.9%	70.4%	108.84	20.20%	0
2019	160,993	30,266	10,343	64.25	108	0.671	10,451	0	10,451	34.5%	100.0%	64.92	18.80%	0
*Paktika*														
2012	407,100	10,362	1025	2.52	69	0.169	1094	12,980	14,074	10.6%	7.8%	34.57	2.55%	2
2013	420,700	16,120	1351	3.21	59	0.140	1410	14,105	15,515	8.7%	9.1%	36.88	3.83%	2
2014	427,692	22,779	2251	5.26	131	0.306	2382	18,568	20,950	10.5%	11.4%	48.98	5.33%	0
2015	434,742	24,769	2040	4.69	177	0.407	2217	15,052	17,269	9.0%	12.8%	39.72	5.70%	0
2016	441,883	27,688	2046	4.63	261	0.591	2307	5690	7997	8.3%	28.8%	18.10	6.27%	1
2017	449,116	37,823	5471	12.18	651	1.450	6122	6379	12,501	16.2%	49.0%	27.83	8.42%	0
2018	748,910	39,002	5621	7.51	550	0.734	6171	3469	9640	15.8%	64.0%	12.87	5.21%	0
2019	762,108	33,888	3063	4.02	164	0.215	3227	3	3230	9.5%	99.9%	4.24	4.45%	0
*Paktya*														
2012	516,300	13,839	1060	2.05	23	0.045	1083	7823	8906	7.8%	12.2%	17.25	2.68%	0
2013	525,500	10,437	916	1.74	53	0.101	969	5551	6520	9.3%	14.9%	12.41	1.99%	0
2014	542,896	14,974	1405	2.59	110	0.203	1515	3751	5266	10.1%	28.8%	9.70	2.76%	0
2015	551,987	13,276	1380	2.50	38	0.069	1418	3022	4440	10.7%	31.9%	8.04	2.41%	0
2016	532,780	13,108	1288	2.42	93	0.175	1381	1131	2512	10.5%	55.0%	4.71	2.46%	0
2017	570,534	20,717	1678	2.94	117	0.205	1795	1112	2907	8.7%	61.7%	5.10	3.63%	0
2018	590,668	17,274	897	1.52	25	0.042	922	506	1428	5.3%	64.6%	2.42	2.92%	0
2019	601,230	12,690	476	0.79	14	0.023	490	0	490	3.9%	100.0%	0.81	2.11%	0
*Panjsher*														
2012	143,700	4242	65	0.45	0	0.000	65	349	414	1.5%	15.7%	2.88	2.95%	0
2013	137,700	3895	20	0.15	0	0.000	20	239	259	0.5%	7.7%	1.88	2.83%	0
2014	151,004	2565	21	0.14	0	0.000	21	110	131	0.8%	16.0%	0.87	1.70%	0
2015	153,487	2106	18	0.12	0	0.000	18	149	167	0.9%	10.8%	1.09	1.37%	0
2016	144,535	1227	10	0.07	3	0.021	13	68	81	1.1%	16.0%	0.56	0.85%	0
2017	158,548	1290	47	0.30	1	0.006	48	46	94	3.7%	51.1%	0.59	0.81%	0
2018	164,115	1273	72	0.44	18	0.110	90	15	105	7.1%	85.7%	0.64	0.78%	0
2019	167,000	928	72	0.43	2	0.012	74	0	74	8.0%	100.0%	0.44	0.56%	0
*Parwan*														
2012	620,900	3276	12	0.02	8	0.013	20	1316	1336	0.6%	1.5%	2.15	0.53%	1
2013	642,300	2803	36	0.06	0	0.000	36	497	533	1.3%	6.8%	0.83	0.44%	0
2014	653,362	2518	33	0.05	0	0.000	33	519	552	1.3%	6.0%	0.84	0.39%	0
2015	664,502	2986	54	0.08	0	0.000	54	510	564	1.8%	9.6%	0.85	0.45%	0
2016	675,795	2516	132	0.20	1	0.001	133	588	721	5.3%	18.4%	1.07	0.37%	0
2017	687,243	2883	440	0.64	20	0.029	460	304	764	16.0%	60.2%	1.11	0.42%	0
2018	711,621	3705	597	0.84	1	0.001	598	35	633	16.1%	94.5%	0.89	0.52%	0
2019	724,561	2407	191	0.26	2	0.003	193	0	193	8.0%	100.0%	0.27	0.33%	0
*Samangan*														
2012	362,500	299	22	0.06	2	0.006	24	540	564	8.0%	4.3%	1.56	0.08%	0
2013	335,700	242	10	0.03	0	0.000	10	424	434	4.1%	2.3%	1.29	0.07%	0
2014	381,459	1697	27	0.07	0	0.000	27	835	862	1.6%	3.1%	2.26	0.44%	0
2015	387,928	2049	4	0.01	0	0.000	4	681	685	0.2%	0.6%	1.77	0.53%	0
2016	394,487	1195	14	0.04	26	0.066	40	885	925	3.3%	4.3%	2.34	0.30%	0
2017	401,134	2066	9	0.02	0	0.000	9	108	117	0.4%	7.7%	0.29	0.52%	0
2018	415,343	1058	2	0.00	1	0.002	3	184	187	0.3%	1.6%	0.45	0.25%	0
2019	422,859	883	4	0.01	0	0.000	4	0	4	0.5%	100.0%	0.01	0.21%	0
*Sar-e-Pul*														
2012	522,900	1341	58	0.11	1	0.002	59	4056	4115	4.4%	1.4%	7.87	0.26%	0
2013	451,000	1591	92	0.20	3	0.007	95	2875	2970	6.0%	3.2%	6.59	0.35%	0
2014	550,238	1442	49	0.09	6	0.011	55	1922	1977	3.8%	2.8%	3.59	0.26%	0
2015	559,577	6391	105	0.19	13	0.023	118	1367	1485	1.8%	7.9%	2.65	1.14%	0
2016	569,043	1708	8	0.01	5	0.009	13	919	932	0.8%	1.4%	1.64	0.30%	0
2017	578,639	3392	8	0.01	1	0.002	9	288	297	0.3%	3.0%	0.51	0.59%	0
2018	599,137	2027	4	0.01	2	0.003	6	143	149	0.3%	4.0%	0.25	0.34%	0
2019	609,986	4546	6	0.01	0	0.000	6	0	6	0.1%	100.0%	0.01	0.75%	0
*Takhar*														
2012	917,700	9009	140	0.15	1	0.001	141	16,297	16,438	1.6%	0.9%	17.91	0.98%	0
2013	950,100	8109	178	0.19	4	0.004	182	11,950	12,132	2.2%	1.5%	12.77	0.85%	0
2014	966,576	22,810	592	0.61	37	0.038	629	4789	5418	2.8%	11.6%	5.61	2.36%	0
2015	983,336	19,671	394	0.40	36	0.037	430	3011	3441	2.2%	12.5%	3.50	2.00%	0
2016	1,000,336	12,812	482	0.48	25	0.025	507	3400	3907	4.0%	13.0%	3.91	1.28%	0
2017	1,017,575	21,014	562	0.55	11	0.011	573	316	889	2.7%	64.5%	0.87	2.07%	0
2018	1,053,852	24,713	410	0.39	2	0.002	412	17	429	1.7%	96.0%	0.41	2.35%	0
2019	1,073,319	21,326	471	0.44	1	0.001	472	0	472	2.2%	100.0%	0.44	1.99%	0
*Uruzgan*														
2012	328,000	4564	110	0.34	10	0.030	120	2109	2229	2.6%	5.4%	6.80	1.39%	0
2013	339,200	4534	162	0.48	34	0.100	196	3746	3942	4.3%	5.0%	11.62	1.34%	0
2014	380,469	3170	83	0.22	6	0.016	89	3115	3204	2.8%	2.8%	8.42	0.83%	0
2015	386,818	4394	95	0.25	15	0.039	110	2414	2524	2.5%	4.4%	6.53	1.14%	0
2016	343,069	4440	98	0.29	5	0.015	103	832	935	2.3%	11.0%	2.73	1.29%	0
2017	362,253	5275	53	0.15	5	0.014	58	889	947	1.1%	6.1%	2.61	1.46%	0
2018	361,030	7134	111	0.31	22	0.061	133	966	1099	1.9%	12.1%	3.04	1.98%	0
2019	428,466	7264	69	0.16	9	0.021	78	0	78	1.1%	100.0%	0.18	1.70%	0
*Wardak*														
2012	558,400	3907	232	0.42	4	0.007	236	979	1215	6.0%	19.4%	2.18	0.70%	0
2013	577,100	3660	160	0.28	4	0.007	164	543	707	4.5%	23.2%	1.23	0.63%	0
2014	586,623	3788	329	0.56	13	0.022	342	753	1095	9.0%	31.2%	1.87	0.65%	0
2015	596,287	5311	449	0.75	17	0.029	466	873	1339	8.8%	34.8%	2.25	0.89%	0
2016	606,077	5052	536	0.88	12	0.020	548	364	912	10.8%	60.1%	1.50	0.83%	0
2017	615,992	6294	589	0.96	31	0.050	620	252	872	9.9%	71.1%	1.42	1.02%	0
2018	637,634	4622	776	1.22	14	0.022	790	31	821	17.1%	96.2%	1.29	0.72%	0
2019	648,866	4035	359	0.55	20	0.031	379	8	387	9.4%	97.9%	0.60	0.62%	0
*Zabul*														
2012	284,600	13,511	2133	7.49	57	0.200	2190	6460	8650	16.2%	25.3%	30.39	4.75%	0
2013	294,100	12,447	1677	5.70	25	0.085	1702	6241	7943	13.7%	21.4%	27.01	4.23%	0
2014	299,125	21,899	554	1.85	46	0.154	600	4117	4717	2.7%	12.7%	15.77	7.32%	0
2015	304,126	15,338	321	1.06	3	0.010	324	2894	3218	2.1%	10.1%	10.58	5.04%	0
2016	309,192	25,039	530	1.71	43	0.139	573	1133	1706	2.3%	33.6%	5.52	8.10%	0
2017	314,325	15,440	191	0.61	14	0.045	205	1042	1247	1.3%	16.4%	3.97	4.91%	0
2018	371,043	15,776	278	0.75	6	0.016	284	556	840	1.8%	33.8%	2.26	4.25%	0
2019	377,648	10,405	697	1.85	40	0.106	737	2	739	7.1%	99.7%	1.96	2.76%	0
